# Biogeographical factors determining *Triatoma recurva* distribution in Chihuahua, México, 2014

**DOI:** 10.7705/biomedica.5076

**Published:** 2020-06-30

**Authors:** María Elena Torres, Hugo Luis Rojas, Luis Carlos Alatorre, Luis Carlos Bravo, Mario Iván Uc, Manuel Octavio González, Lara Cecilia Wiebe, Alfredo Granados

**Affiliations:** 1 Unidad Multidisciplinaria, Universidad Autónoma de Ciudad Juárez, Cuauhtémoc, Chihuahua, México Universidad Autónoma de Ciudad Juárez CuauhtémocChihuahua México; 2 Instituto de Ingeniería y Tecnológica, Departamento de Ingeniería Civil y Ambiental, Juárez, Chihuahua, México Instituto de Ingeniería y Tecnológica JuárezChihuahua México

**Keywords:** Triatoma, Triatominae, ecosystem, Chagas' disease, disease vectors, climate, Triatoma, Triatominae, ecosistema, enfermedad de Chagas, vectores de enfermedades, clima

## Abstract

**Introduction:**

*Triatoma recurva* is a *Trypanosoma cruzi* vector whose distribution and biological development are determined by factors that may influence the transmission of trypanosomiasis to humans.

**Objective:**

To identify the potential spatial distribution of *Triatoma recurve,* as well as social factors determining its presence.

**Materials and methods:**

We used the MaxEnt software to construct ecological niche models while bioclimatic variables (WorldClim) were derived from the monthly values of temperature and precipitation to generate biologically significant variables. The resulting cartography was interpreted as suitable areas for *T. recurva* presence.

**Results:**

Our results showed that the precipitation during the driest month (Bio 14), the maximum temperature during the warmest month (Bio 5), and the altitude (Alt) and mean temperature during the driest quarter (Bio 9) determined *T. recurva* distribution area at a higher percentage evidencing its strong relationship with domestic and surrounding structures.

**Conclusions.:**

This methodology can be used in other geographical contexts to locate potential sampling sites where these triatomines occur.

## Resumen

**Introducción.:**

*Triatoma recurva* es un vector de *Trypanosoma cruzi* cuya existencia y desarrollo biológico están determinados por factores que pueden influir en la transmisión de la tripanosomiasis a los seres humanos.

**Objetivo.:**

Determinar una posible distribución espacial de *Triatoma recurva* y algunos factores sociales que determinan su presencia.

**Materiales y métodos.:**

El modelado de nicho ecológico se hizo con el programa MaxEnt empleando las variables bioclimáticas (WorldClim) derivadas de los valores mensuales de temperatura y precipitación para generar variables biológicamente significativas. La cartografía resultante evidenció áreas adecuadas para la presencia de *T. recurva*.

**Resultados.:**

Los resultados indicaron que la precipitación del mes más seco (Bio 14), la temperatura máxima del mes más cálido (Bio 5), y la altitud (Alt) y la temperatura media del trimestre más seco (Bio 9), determinaron en mayor porcentaje el área de distribución de *T. recurva*, observándose que es una especie con una acentuada relación con las estructuras domésticas y circundantes.

**Conclusión.:**

Esta metodología puede emplearse en otros contextos geográficos para localizar posibles sitios de muestreo de estos triatominos.

Chagas disease or American trypanosomiasis is a parasitic blood and tissue disease caused by the flagellated protozoan *Trypanosoma cruzi* located in tissue, especially myocardial tissue. After a long evolutionary period ^1^^,^^2^, *T. cruzi* is known to cause irreversible heart disease in 25 % of affected people. Its transmission cycle constitutes a complex zoonosis that involves several vertebrate reservoirs and insects among which *Triatoma recurva* is one of the chief ones. *Trypanosoma cruzi* can infect many tissues of mammalian hosts and spreads in the environment in multiple transmission cycles that may or may not be connected ^3^^,^^4^.

These insects belong to the order Hemiptera, infraorder Cimicomorpha, superfamily Reduvioidae, family Reduviidae, and subfamily Triatominae comprising more than 140 species grouped into 18 genera and five tribes. The habitats for their life cycles show considerable variability, and authors have indicated that some triatomine species can adapt to home environments and their peripheries where they transmit *T. cruzi* to humans ^5^.

Chagas disease is the most important parasitic disease in Latin America given its morbidity and economic importance. It surpasses all other parasitic diseases ^6^ and is the third most infectious disease in Latin America, second only to AIDS and tuberculosis. In Mexico, this type of zoonosis is associated with people's socio-economic level, which determines their access to resources, hygiene practices, and quality of housing, education, and sanitation, especially potable water and drainage systems ^7^^,^^8^. *Triatoma recurva* represents a risk factor for the population at large ^9^^,^^10^ but access to information about this type of zoonosis in nonendemic places is insufficient because the disease is not considered a risk and record-keeping of any related data is scant. Additionally, the gathering of epidemiological data regarding the death toll from Chagas disease is challenging ^11^ due to the lack of experience in its clinical diagnosis, which also affects decision making in medical surveillance limited at best.

Factors such as climate, rain, geographical barriers, humidity, topography, hosts, reservoirs, and causal agents determine *T. recurva* distribution and, therefore, its ability to transmit *T. cruzi*^12^^-^^14^. The knowledge about the spatial location of these factors and the distribution of these Hemiptera can be extremely useful to detect populations vulnerable to diseases transmitted by these vectors.

Geographic Information Systems have become an epidemiological tool to monitor vector-transmitted diseases ^15^^-^^17^ and develop proper intervention strategies. To identify the biophysical variables required by a taxonomic group, Phillips, *et al.* developed a maximum entropy algorithm known as MaxEnt, which combines statistics and Bayesian methodology to estimate the distributions of the maximum entropy subject to environmental information constraints ^18^.

The geographical distribution of *T. recurva* is essential to study its natural, ecological, genetic, and evolutionary history, as well as to obtain the information needed to understand the different biogeographical and historical factors conditioning the different diseases it transmits. Such knowledge would contribute as well to foresee a potential emergence or reemergence of diseases transmitted by the vector and to extend the current view of the distribution of this important group of insects in México. In this context, the objective of our study was to identify the potential spatial distribution of *T. recurva* and the factors determining its presence.

## Materials and methods

### Study area

**Figure 1:**
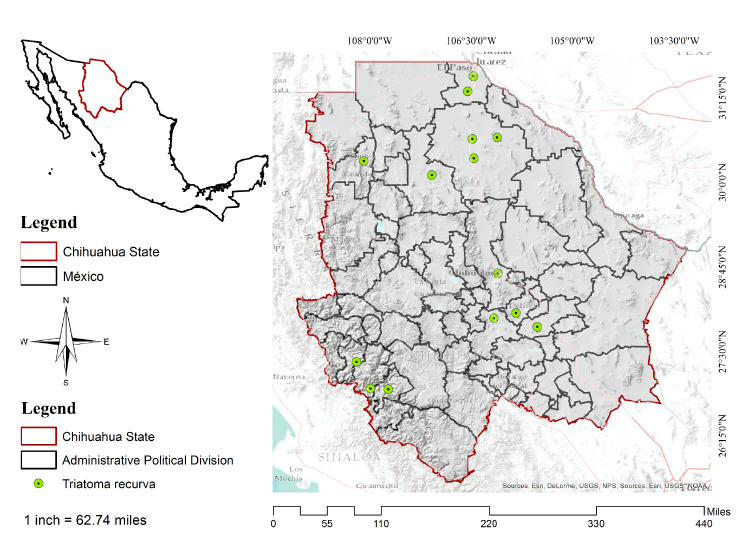
Area of study and presence of *Triatoma recurva*

Chihuahua is in the central part of northern México (figure 1) bordering in the north the states of New Mexico and Texas in the United States, Coahuila de Zaragoza in the east, Durango state in the south, and Sinaloa state in the west. Its geographical coordinates are 25°30' and 31°47' N and 103°18 to 109°07 W. It is México's largest state stretching across 12% of the nation's surface with a total area of 247.45 km^2^. Its climate is dry and semidry and the rainfall annual average is approximately 500 mm ^19^.

### Database and procedures

To generate the area of *T. recurva* potential distribution, we used 14 records of occurrence, all of them located in the state of Chihuahua. The points of presence were taken from the Global Biodiversity Information online website ^1^, Licón-Trillo ^1^, and the *Centro Nacional de Programas Preventivos y Control de Enfermedades* (Cenaprece) ^12^^,^^20^^-^^23^.

We used a group of biophysical variables: 19 climatological and five topographical derived from the digital elevation model (hillshade), accumulation of flux, slope, hillside facings, and altitude (Z) obtained from WorldClim (table 1). This database contains climate data corresponding to global climate layers with a homogenized resolution of 1 km obtained by cross-referencing weather station records (grids of 20 x 20 km, Environmental Systems Research Institute - ESRI format) from 1950 to 2000 from several sources at the global, national, regional, and local levels. These layers feature bioclimate variables derived from the monthly temperature and rainfall values to generate the most biologically significant variables representing annual tendencies and limiting factors for the species distribution ^20^.

**Table 1:** Environmental parameters for species distribution models: WordClim (1950-2000), NDVI-MODIS 2014, topographic, and land-use variables

Variable	Description
BIO1	Annual mean temperature
BIO2	Mean diurnal range**
BIO3	Isothermality
BIO4	Temperature seasonality
BIO5	Maximum temperature of warmest month**
BIO6	Minimum temperature of the coldest month
BIO7	Temperature annual range**
BIO8	Mean temperature of the wettest quarter (3 months)
BIO9	The mean temperature of the driest quarter (3 months)**
BIO10	Mean temperature of the warmest quarter (3 months)
BIO11	Mean temperature of the coldest quarter (3 months)
BIO12	Annual precipitation
BIO13	Precipitation of wettest month
BIO14	Precipitation of driest month**
BIO15	Precipitation seasonality
BIO16	Precipitation of wettest quarter (3 months)
BIO17	Precipitation of driest quarter (3 months)
BIO18	Precipitation of warmest quarter (3 months)
BIO19	Precipitation of coldest quarter (3 months)
NDVI1	Normalized difference vegetation index to January, 2014
NDVI2	Normalized difference vegetation index to February, 2014
NDVI3	Normalized difference vegetation index to March, 2014
NDVI4	Normalized difference vegetation index to April, 2014
NDVI5	Normalized difference vegetation index to May, 2014
NDVI6	Normalized difference vegetation index to June, 2014
NDVI7	Normalized difference vegetation index to July, 2014
NDVI8	Normalized difference vegetation index to August, 2014
NDVI9	Normalized difference vegetation index to September, 2014
NDVI10	Normalized difference vegetation index to October, 2014
NDVI11	Normalized difference vegetation index to November, 2014
NDVI12	Normalized difference vegetation index to December, 2014
Alt	Altitude Z**
Acu	Accumulation of flux**
Pend	Slope
Asp	Situation of hillsides
Somb	Hillshade**
Uso	Soil use**

** Variables used for the model were obtained (not related among them) from Spearman's correlation (<0.75, p=95%, a=0.025) and bootstrap of 1000 iterations.

We complemented this information with the vegetation tables of contents from the Normalized Difference Vegetation Index (NDVI) generated by the NASA MODIS sensor in 2014 ^21^^-^^23^. These tables have a temporary monthly resolution and a spatial resolution of 1 km^2^ (12 monthly NDVI variables by the 11th of each month) (MODIS. GSFC. NASA. gov/data-dataprod-mod13. php), as well as the land-use variable generated by the *Instituto Nacional de Estadística y Geografía* (INEGI) for land use and the V 2015 vegetation series available in the vectorial format at a 1:250,000 scale in the geosite of the *Comisión Nacional para el Conocimiento y Uso de la Biodiversidad* (CONABIO) with 37 variables in total ^24^.

### Adjustment of the spatial resolution

As the information collected was generated using different scales, it was necessary to standardize the scales based on the characteristics of the WorldClim variables (1 km given that MaxEnt does not work at different scales) (columns, rows, and pixel size) using ArcGis 10X with extraction using the mask module ^25^.

### Selection of variables

The first step was to analyze the spatial correlation of the 37 predictor variables in the study area. For this, we calculated Spearman's correlation between pairs of variables that ruled out correlation values exceeding R>0.75 because 0.5 to 0.7 coefficients tend to be relevant in small samples and this type of correlation avoids the oversizing of presence areas and should be used for data series with extreme values because Pearson's correlation calculations will affect the results ^26^^,^^27^.

Additionally, we made a bootstrap resampling (1,000 repetitions) where the independent covariables were expected to be present in the largest number of bootstrap samples while noise variables were present as predictors in a lesser number of bootstrap samples ^28^. If it is carried out automatically, the advantage of this resampling technique is that it allows the estimation of an empirical distribution function through the resampling of the observed data and autocorrelation does not affect the selected model (table 1) ^29^.

The standard deviation was calculated with a confidentiality interval bias at 95% and a level of significance of α=0.025 using the IBM SPSS Statistics™, version 20.0, software. This process yielded nine representative variables for the area of interest. Using the jackknife procedure we eliminated negative contributions and, thus, three variables were eliminated (Bio2, Somb, and Uso).

### Potential distribution

First, it was necessary to debug the database with occurrence records. Each point records information on its location, i.e., latitude and longitude in decimal degrees. The preparation of the environmental variables comprised setting the type of format to ASCII because MaxEnt only recognizes this format and geospatially adjusting each variable to the study area.

We selected the MaxEnt algorithm because its application in previous works had yielded good results ^30^^,^^31^ even with scant data ^32^, as in this case. Usually, in MaxEnt, the data are divided into two sets, one for the generation of the model and the other one for validation ^33^^,^^34^. However, as this procedure loses important information within the data set for validation ^35^, it is not suitable with small samples ^35^ but to solve the problem we resorted to a replication technique (bootstrapping) and generated 50 models. In this way, random partitions of data were made in each replication and each model was valued using a user-defined percent (50% in this case). In bootstrapping, sampling is performed by replacement indicating that the records of presence can be used more than one time in the validation dataset for each replication ^36^^-^^38^.

The biophysics variables were of the continuous type in our case. To estimate which variables were more relevant to the model, we discarded the variables that did not contribute to it using a jackknife test and then, the test was performed again with the newly debugged data ^39^.

The logistic output was chosen to obtain those values that were easier to understand and were processed later to be used as a probability value with values fluctuating between 0 and 1 where 0 showed incompatibility or absence of the species and 1, suitability, or likelihood of the species presence ^40^.

The evaluation process followed the parameters established by Phillips, *et al.*^31^, using the characteristic receiver's operating curve (ROC) to calculate the area under the curve (AUC), which was obtained by comparing the proportion of false and true positives, i.e., to show in two axes, X and Y, the proportion of false positives (1-specificity) and on the Y-axis the proportion of true positives (sensitivity) ^41^. An AUC with a 0.5 value shows that the model has no predictive power, a value of 1 shows discrimination or a perfect model, and values below 0.5 show a much lesser relationship than that randomly expected ^42^^,^^43^.

After generating the 50 models, we selected five maps from MaxEnt, specifically those with a greater area under the curve to incorporate them into the ESRI ArcGis, 10.2 version. Through map algebra, we calculated the average of such selection to obtain a consensus map and define the potentially sustainable areas for the species. Then we reclassified the values based on the threshold established by the MaxEnt: 10 percentile (a 10% probability that the points of presence lied out of the prediction area of the whole potential distribution area) ^44^. Probabilities under the threshold were transformed into 0 and interpreted as the absence of hemipteran while those over the threshold were converted to 1 showing the presence of the species.

## Results

The models, based on random subsets, were highly predictive of the distribution of *T. recurva*. The AUC results and highest percentages of the contribution of the two-variable model by replication showed sustained importance of the following variables (table 2): precipitation during the driest month, the maximum temperature of the warmest month, altitude (Alt), and mean temperature of the driest quarter, all indicating that the model ability to classify the presence was good and that it can be considered acceptable and more precise than that of a randomly obtained model.

**Table 2:** Area under the curve and highest percentage contribution of variables by reply

Number of models	AUC	Variable of importance	Percentage contribution
17	0.8526	Precipitation of driest month	72.1
		Max temperature of warmest month	27.9
16	0.8449	Max temperature of warmest month	89.6
		Precipitation of driest month	10.4
26	0.8243	Max temperature of warmest month	58.5
		Precipitation of driest month	41.5
40	0.8145	Precipitation of driest month	99.4
		Altitude	0.6
6	0.8136	Mean temperature of driest quarter	100
Number of models	AUC	Variable of importance	Percentage contribution
17	0.8526	Precipitation of driest month	72.1
		Max temperature of warmest month	27.9
16	0.8449	Max temperature of warmest month	89.6
		Precipitation of driest month	10.4
26	0.8243	Max temperature of warmest month	58.5
		Precipitation of driest month	41.5
40	0.8145	Precipitation of driest month	99.4
		Altitude	0.6
6	0.8136	Mean temperature of driest quarter	100

AUC: area under the curve

**Figure 2:**
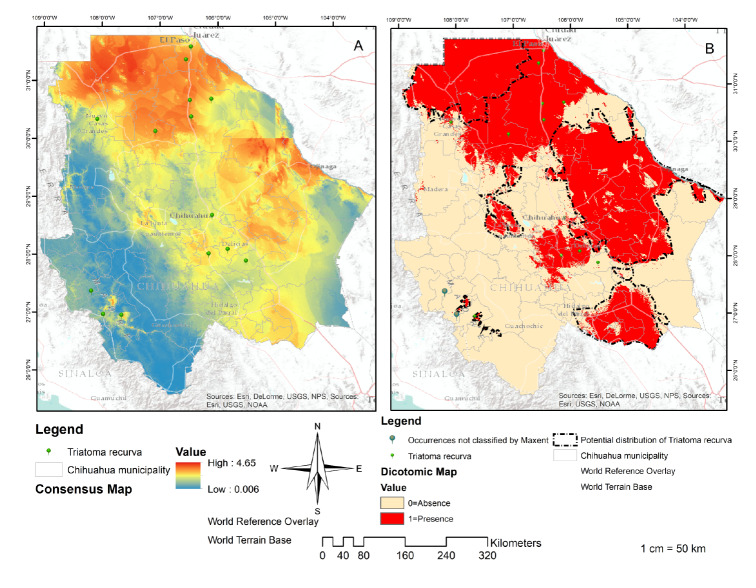
Distribution of *Triatoma recurva*. Consensus map **(A):** Result of the addition of the five models with AUC<0.8. Source: Individual elaboration based on the results obtained by the modeling in MaxEnt and algebra of maps. Map of presence/absence **(B)** for the *Triatoma recurva* generated by the reclassification and algebra of maps. Source: Individual elaboration based on the results of MaxEnt, the reclassification by the percentile 10, and algebra of maps. The dotted line shows favorable environmental conditions for the presence of *Triatoma recurve* in Chihuahua state (potential distribution). There are also two occurrences not classified by Maxent.

The resulting cartography was obtained through the replicas ^6^^,^^16^^,^^17^^,^^26^^,^ and ^40^ with an AUC more significant than 0.8 (figure 2). Table 2 shows the percentage contribution of the variables used to build the potential distribution model for *T. recurve,* the mean temperature of the driest quarter (Bio 9) being the most critical variable for its distribution (100% contribution), followed by the highest temperature of the warmest month (Bio 5), the precipitation of the driest month (Bio 14) (99.4%) and the altitude (Alt) (0.6%).

**Figure 3:**
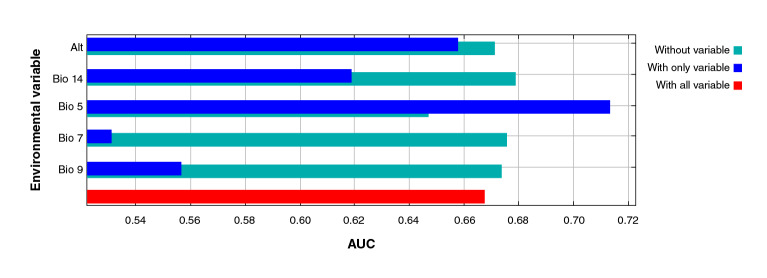
Jackknife test of the area under the curve (AUC) for *Triatoma recurva*. Profit generated by each variable in three different scenarios: (a) running the model with only one variable (blue); (b) with all the variables except one (green), and (c) with all the variables (red). This reflects how much useful information each variable contains.

However, the jackknife test indicated the variables bringing more information to the model when isolated: Bio 5 (maximum temperature of the warmest month), altitude (Alt), precipitation of the driest month (Bio 14), mean temperature of the driest quarter (Bio 9), and annual temperature range (Bio 7) (figure 3).

The resulting cartography can be interpreted as moderate to highly suitable areas in the municipalities of Buenaventura, Galeana, Ahumada Casas Grandes, Praxedis G. Guerrero, Batopilas, Urique, Morelos, Guachochi, Ascension, Ojinaga, Coyame del Sotol, Aldama, Manuel Benavides, Julimes, Delicias, Rosales, Saucillo, Meoqui, La Cruz, Allende Hidalgo del Parral, Matamoros, and Coronado.

## Discussion

The biogeographical approximation we adopted in the present study was based on the potential distribution of *T. recurva*, which is the only component involved in the vectorial transmission dynamics of Chagas disease, a zoonosis that must be understood as an extremely complex natural system.

The values of the area under the curve (greater than 0.8) in the *T. recurva* models were above the random prediction parameter (AUC=0.50) indicating that the model's ability to classify the presence of the species is acceptable and more precise than a randomly obtained model.

Additionally, this result is consistent with reports from other authors 45-49. MaxEnt was proven to be a useful tool for modeling using only the data of presence, making predictions with low numbers of presence, and improving the performance of numerous traditional techniques.

Regarding the AUC jackknife test, we noticed that Bio 5 (highest temperature of the warmest month) is the highest-contributing variable to the isolated model form, followed by the mean temperature of the driest quarter (Bio 9), the altitude (Alt), the precipitation of the driest month (Bio 14), and the annual temperature range (Bio 7). These five variables bear a relationship to each other and explain why several authors consider them as constraining factors for this species ^50^^-^^54^.

The cartography resulting from the MaxEnt model can be interpreted as the potential areas in the above-mentioned municipalities and they explain why *T. recurva* has a higher probability of existing in those places due to their environmental conditions. However, triatomine bugs could be present in all the northern cone of Chihuahua (figure 2A) 55,56. The map of the absence or presence defines the potential distribution of *T. recurva* (figure 2B) but its biological and geographical information is limited because this species is rarely collected and its breeding in the laboratory is challenging ^57^.

Two omissions (figure 2B) can be attributed to the generated model, which will not be accurate given that the occurrences were non-representative, to a misidentification of species, georeferencing, or to the fact that the presence of the species include individuals outside their native distribution ^58^. However, some authors have indicated that in some models small areas contain the corresponding species showing that they are not absent but that their distribution has lost continuity in these areas and generated isolated populations due to the historical or recent fragmentation of their habitat.

The data previously mentioned show *T. recurva*'s capacity of adjustment to different environments. In the present study, we identified possible patterns of the distribution of medically important Hemiptera. Among the 37 variable predictors used in the modeling, four predicted the potential distribution of *T. recurva* (mean temperature of the driest quarter, Bio 9; the maximum temperature of the warmest month, Bio 5; precipitation of the driest month, Bio 14, and altitude, Alt) ^45^^,^^50^^-^^53^. The cartography of the potential spatial distribution of *T. recurva* was generated through modeling using maximum entropy.

Given the resurgence of diseases transmitted by Hemiptera, these results can be helpful to generate a hypothesis and identify critical locations where diseases caused by this vector spread and are transmitted. The AUC values reached indicated that the model predicts the distribution of triatomine bugs in Chihuahua, México, with a very acceptable degree of precision, higher than those obtained randomly, thus confirming our findings and the validity of the model obtained.

More attention should be given to the variables that intervene importantly in the generation of this model because they indicate the presence of Hemiptera and allow for the development of control and sanitation strategies to avoid epidemics in the country. The knowledge on the biogeographical process of vectors such as Hemiptera is essential for the development, planning, and optimization of preventive actions and vector control. The modeling of the potential occurrence of *T. recurva* is an approach to identify the vulnerable zones in the country and they should be considered valid tools.

More in-depth studies are required to develop government programs for the control of vector-borne diseases.

The present study contributes basic information to feed the nation's epidemiological surveillance system focusing on those states where the suitability map has the highest values or where sub-records indicate the presence of these vectors. This methodology can be used in other geographical contexts to locate potential sampling sites where these triatomines occur.
